# Mathematical modelling as a proof of concept for MPNs as a human inflammation model for cancer development

**DOI:** 10.1371/journal.pone.0183620

**Published:** 2017-08-31

**Authors:** Morten Andersen, Zamra Sajid, Rasmus K. Pedersen, Johanne Gudmand-Hoeyer, Christina Ellervik, Vibe Skov, Lasse Kjær, Niels Pallisgaard, Torben A. Kruse, Mads Thomassen, Jesper Troelsen, Hans Carl Hasselbalch, Johnny T. Ottesen

**Affiliations:** 1 Department of Science and Environment, Roskilde University, Roskilde, Denmark; 2 Department of Laboratory Medicine at Boston Children’s Hospital, Boston, Massachusetts, United States of America; 3 Department of Hematology, Zealand University Hospital, University of Copenhagen, Roskilde, Denmark; 4 Department of Pathology, Zealand University Hospital, University of Copenhagen, Roskilde, Denmark; 5 Department of Clinical Genetics, Odense University Hospital, Odense, Denmark; Holbæk Hospital, DENMARK

## Abstract

The chronic Philadelphia-negative myeloproliferative neoplasms (MPNs) are acquired stem cell neoplasms which ultimately may transform to acute myelogenous leukemia. Most recently, chronic inflammation has been described as an important factor for the development and progression of MPNs in the biological continuum from early cancer stage to the advanced myelofibrosis stage, the MPNs being described as “A Human Inflammation Model for Cancer Development“. This novel concept has been built upon clinical, experimental, genomic, immunological and not least epidemiological studies. Only a few studies have described the development of MPNs by mathematical models, and none have addressed the role of inflammation for clonal evolution and disease progression. Herein, we aim at using mathematical modelling to substantiate the concept of chronic inflammation as an important trigger and driver of MPNs.The basics of the model describe the proliferation from stem cells to mature cells including mutations of healthy stem cells to become malignant stem cells. We include a simple inflammatory coupling coping with cell death and affecting the basic model beneath. First, we describe the system without feedbacks or regulatory interactions. Next, we introduce inflammatory feedback into the system. Finally, we include other feedbacks and regulatory interactions forming the inflammatory-MPN model.

Using mathematical modeling, we add further proof to the concept that chronic inflammation may be both a trigger of clonal evolution and an important driving force for MPN disease progression. Our findings support intervention at the earliest stage of cancer development to target the malignant clone and dampen concomitant inflammation.

## Introduction

The classic chronic Philadelphia-negative myeloproliferative neoplasms (MPNs) include essential thrombocythemia (ET), polycythemia vera (PV) and primary myelofibrosis (PMF), which are acquired stem cell neoplasms [1]. Most patients live with their MPNs for decades although with a huge morbidity burden due to a high risk of thrombosis with cardiovascular complications and a massive comorbidity burden as well due to an increased propensity to develop autoimmune and chronic inflammatory diseases [2–4], including a 40% increased risk of second cancers [5,6]–not only after the MPN-diagnosis but also prior to the MPN-diagnosis [7]. Several years prior to the MPN-diagnosis these patients also have an increased risk of cardiovascular, autoimmune and inflammatory diseases [8,9]. Furthermore, the MPNs have an inherent risk of transformation to acute myelogenous leukemia (AML) and myelodysplastic syndrome (MDS) [10].

During the last decade major breakthroughs have occurred in the understanding of the pathogenesis of the MPNs, the most important being the identification of the somatic clonal markers–JAK2, MPL and CALR [11–18]. The findings of several other mutations already at the time of MPN-diagnosis, with the emergence of additional mutations in the advanced transforming stages of MPNs [17,18], all support the concept of a biological continuum from the early cancer stages (ET/PV) to the advanced cancer stages (myelofibrosis or AML) [1,19,20]. Chronic inflammation is the common link between common diseases such as atherosclerosis, the metabolic syndrome, type II diabetes mellitus and cancer, in which the JAK-STAT- signalling and the NF-kB pathways are activated and have major roles in disease progression [21–28]. These pathways are activated in MPNs as well. Most recently, the MPNs have been described as “Inflammatory Diseases “[4] and “A Human Inflammation Model For Cancer Development”[29] reflecting chronic inflammation to be a major driving force for clonal evolution and disease progression in MPNs [30–39]. This novel concept is built upon a platform, which has combined data from studies in several research fields and disciplines within MPNs—clinical [3–9,29–53], experimental [54–63], genomic [64–70], immunological [71–74] and not least epidemiological studies [3,5–7,75–77].

Another research field—mathematical modelling of cancer development—has not been applied to a similar extent within MPNs until very recently [78,79] and not in the context of investigating the concept of MPNs as “A Human Inflammation Model for Cancer Development”. Mathematical modelling of cancer development has provided new insights regarding cancer initiation and progression [80–89]. In this context, mathematical modelling has a huge potential to support or disprove understanding of research data on pathogenetic factors of significance for cancer development but also in regard to providing supportive evidence for a drug to be used in cancer therapy and accordingly a novel tool in evidence-based medicine [90–92]. Mathematical modelling of chronic inflammation as the trigger and driver of MPNs has never been investigated. Although the concept of MPNs as “inflammatory diseases” is being increasingly recognized, additional proof of this novel concept by mathematical modelling might be of utmost importance not only for our understanding of the pathogenesis of these neoplasms, but also in regard to diagnosis and treatment. Herein, we for the first time by mathematical modelling add further proof of the concept that MPNs may be both triggered and driven by chronic inflammation. We discuss the perspectives of our findings, which might implicate intervention at the earliest stage of cancer development (ET, PV) to target the malignant clone and dampen concomitant inflammation when the tumor burden is minimal, and accordingly, the outcome of treatment is logically most favorable.

## Methods

**Fig 1:**
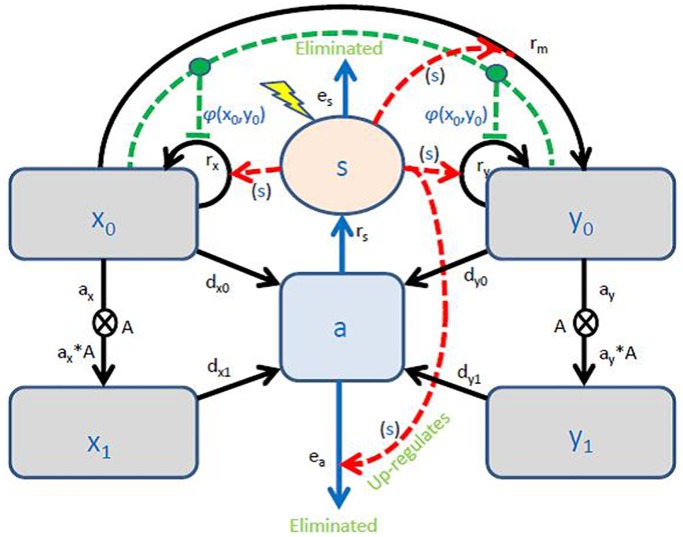
The conceptual model. Light gray boxes (symbolized x_0_, x_1_, y_0_, and y_1_) illustrate the compartments of the basic model, and the black arrows the rates of the flows between these compartments. Here x_0_ denotes the number of HSC, x_1_ that of HMS, y_0_ that of MPN SC, and y_1_ the number of MPN MC. The light blue compartment (symbolized a) contains all dead cells and the light orange compartment (symbolized s) the inflammatory level, i.e. the immune response. Blue arrows from these represent related rates of flows. Red stipulated arrows going from the inflammatory compartment represent effects of the cytokines (or neutrophils when eliminating dead cells) modulating rates of the basic model. Two additional rates (depending on x_0_ and y_0_) appearing as red stipulated arrows represent the bone marrow niches symbiosis with the stem cells modulating the self-renewal rates. Note, stem cells leaving their respective compartments enter the corresponding mature cell-pools as multiplied by the progenitor amplification factor (A).

The system describes the proliferation from stem cells to mature cells including mutations of healthy stem cells to become malignant stem cells. We include regulatory interactions (e.g. niche growth effects) and inflammation coping with cell death, inflammatory cytokines, and neutrophils. In order to design an inflammatory MPN model, we build on the coupled dynamics of inflammation and cancer progression as depicted in Fig 1.

### The model

Most previous studies attempting to model the role of inflammation and immune deregulation in cancer progression consider solid tumors and couple the T-cell and natural killer (NK) cell dynamics to a logistic growth of a tumor. They mainly describe quite simplified versions of the adaptive immune response without explicitly considering the underlying cancer growth dynamics [93–98]. In contrast to all these models, our model is the first which couples the principles underlying actual cell dynamics to a basal inflammatory response. This response is seen for a normal infection, where the amount of dead cells provokes the immune response and stimulates the renewal of stem cells. Despite this complex coupling, the model is kept as simple as possible still allowing the relevant quantities to be described. Thus, the goal is to describe an important coupling between MPN development and the inflammatory response at a quantitatively conceptual level. Hence, the complicated mathematical question of model identifiability and accurate parameter estimation will be addressed elsewhere. Nevertheless, we include some model calibration and validation after presenting the model to justify and demonstrate the strength of the model.

Basically, our model consists of four pools of cells; the hematopoietic stem cells (HSC), the hematopoietic mature cells (HMC), the MPN-mutated stem cells (MPN SC) and the MPN mature cells (MPN MC). The number of these cells are denoted *x*_*0*_, *x*_*1*_, *y*_*0*_, and *y*_*1*_ respectively, where *x* refers to normal hematopoietic cells and *y* to MPN hematopoietic cells, while index *0* refers to stem cells and index *1* to mature cells. A single stem cell (SC) may proliferate in three ways; symmetric self-renewal (having two stem cells as offspring), asymmetric self-renewal (turning into one stem cell and one progenitor cell), and symmetric differentiation (giving rise to two progenitor cells). The progenitor cells cannot be ignored, however, we consider the progenitor cells simply as intermedia multiplication steps describing the way stem cells generate mature cells. In the model, the generations or continuum of progenitor cells will be implicitly accounted for as each stem cell will generate a number of mature cells by an amplification factor, *A (= 2*^*k*^ if there are *k* generations of progenitor cells). Feedbacks from or to progenitor cells are ignored or integrated into the other included feedbacks.

The present focus is on the ensemble of each cell type and not the individual cells; thus the governing laws will be for the pools of cells, in science denoted compartments [99]. Mathematically, the dynamics will be described by non-linear ordinary differential equations respecting conservation laws. The HSC self-renews with rate *r*_*x*_ and the malignant MPN SC self-renews with rate *r*_*y*_. Furthermore, HSC may be transformed by cell division by a rate *a*_*x*_ whereas the MPN SC does so with a rate *a*_*y*_. The mature cells are multiply generated, i.e. the HMC are generated with a rate *a*_*x*_*·A*_*x*_ and the MPN MC with a rate *a*_*y*_*·A*_*y*_. Finally, all cell types may die; stem cells with a lower rate and mature cells with a higher rate. The turnover (or mortality) rates are *d*_*x0*_, *d*_*x1*_, *d*_*y0*_, and *d*_*y1*_ for the HSC, HMC, MPN SC, and MPN MC, respectively. Except for the mutation part and the multiplication factor, this duplicates the structure of the model proposed by Dingli and Michor (they silently used *A = 1)* [92].

A small probability r_m_ describes the mutation of HSC into MPN SC. In that case, r_m_ is not the probability of a single mutation but possibly a serial sequence of mutations turning the HSC into a cancer cell capable of self-renewal, by definition an MPN SC, where a mutation is expected to be described by a Poisson process [100]. The probability for one mutation is about 10^−7^ per year per cell [101]. However, not all mutations are malignant; only mutations which happen on particular locations (i.e. at specific nucleic acids) of the DNA cause MPN relevant mutations. Inflammation increases the risk of mutations, including smoking, exposure to ultraviolet light or certain chemicals [49,50,101–104]. It is this small probability which violates a possible deterministic description with a simple mutation rate. Except for the mutation part, the model will be deterministic and continuous. In most of our work, we studied the development right after the first malignant mutation has occurred (denoted the first insult). In these cases, the simulations start with one malignant stem cell. Meanwhile, the number of all other cells are in a healthy steady state with the mutation rate put to zero. The approach is justified by the fact that including a non-zero mutation rate did not affect the outcome of the model.

The equations are all of the general form,

$$\left\{\begin{array}{c} \text{Change in amount of a }\\ \text{compartment per time} \end{array}\right\}=\left\{\begin{array}{c} \text{rate of generation times }\\ \text{the generating source} \end{array}\right\}-\left\{\begin{array}{c} \text{rate of elimination times the amount }\\ \text{in the compartment considered} \end{array}\right\}$$

resulting in specific systems of ordinary differential equations as given in S1 Appendix.

Whenever cells die the debris have to be engulfed by phagocytic cells, e.g. neutrophils and macrophages while a hierarchic cascade of pro- and anti-inflammatory cytokines are released [96–98,110]. Following the parsimonious principle, we let the dead cells (a) up-regulate the amount of phagocytic cells (*s*) with rate constant *r*_*s*_ per dead cell while they are eliminated with a rate *e*_*s*_. In addition, endotoxins, smoking and other environmental factors may add to the inflammatory response; thus we add such a term (characterized by the lightning symbol in Fig 1). Since MPNs develop on time-scale years and inflammatory immune processes are fast (on time-scale hours-days), we assume that the amount of phagocytic cells is balanced by the cytokines levels in a fixed ratio. Thus, the cytokine level is proportional to the phagocytic level why the inflammatory compartment (*s*) represents both (up to a possible proportionality constant which may be incorporated into the rate constants). Meanwhile, the amount of dead cells is down-regulated as a second order elimination process, *-e*_*a*_*·a·s*, with rate constant *e*_*a*_. Dead cells are produced by *d*_*0*_*·x*_*0*_*+d*_*y0*_*·y*_*0*_*+d*_*x1*_*·x*_*1*_*+d*_*y1*_*·y*_*1*_ per time denoted the turnover, which is assessed by the plasma concentration of lactic dehydrogenase (LDH). It is well-known that the inflammatory level affects the mutation rate [104] and the self-renewal rates [105]. For simplicity, we take these to be proportional with the inflammatory level (of course saturation may occur) but since the level (a) settles at constant levels so does the inflammatory level (s), which may be thought of as the amount of inflammatory cytokines which have been shown to be increased in patients with MPNs and several in a step-wise manner from controls over the early cancer stages (ET, PV) to the advanced cancer stage–myelofibrosis (PMF) (S1 Appendix) [40–46]. Thus, it turns out that various specific cytokines (IL-1β, IL-1RA, IL-2R, IL-6, IL-8, IL-10, IL-12) and C-reactive protein (CRP)—a conventional biomarker of inflammation—are linearly correlated with the inflammatory level (s). These cytokines have been chosen for validation of our model since elevated levels of several of these cytokines have been associated with an inferior survival [44]. Likewise, elevated levels of CRP have been shown to be associated with shortened leukemia-free survival in patients with myelofibrosis [42]. Of note, the inflammatory cytokine IL-8 have been reported to be of particular interest in the context of MPN pathogenesis [57–60]. These extra pools of cells are depicted in Fig 1 along with the rates governing the dynamics. This establishes the coupled inflammatory-MPN model. The full system of mathematical equations, representing the model is described in Table B in S1 Appendix including default parameter values.

### Model calibration, validation, and results

**Fig 2:**
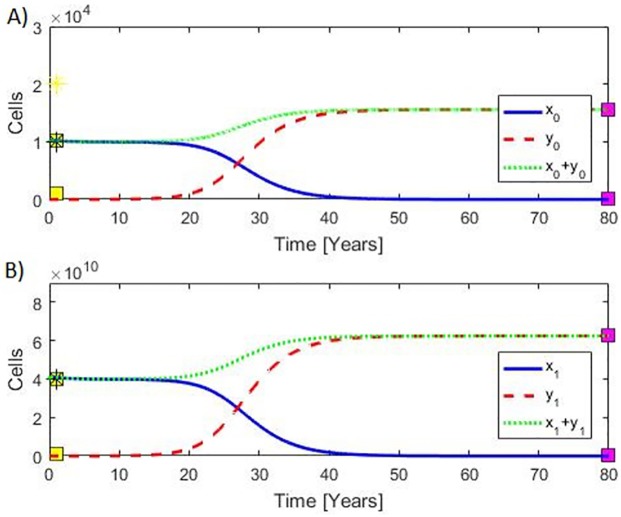
Model calibration. A) Plateaus to the left show the amount of hematopoietic stem cells *x*_0_ (upper plateau) and that for MPN stem cells *y*_0_ (lower plateau) whereas the plateaus to the right show the amount of hematopoietic stem cells *x*_0_(lower plateau) and MPN mature cells *y*_0_ (upper plateau). B) Plateaus to the left show the amount of hematopoietic mature cells *x*_1_ (upper plateau) and that for MPN mature cells *y*_1_ (lower plateau) whereas the plateaus to the right show the amount of hematopoietic mature cells *x*_1_ (lower plateau) and MPN mature cells *y*_1_ (upper plateau). The yellow and purple boxes show our data used for calibrating (and validating) the model with further details in S1 Appendix. Yellow boxes show our “no MPN cancer values”, and purple boxes show our “full blown” MPN values in the advanced myelofibrosis stage. Yellow position marker shows the number of hematopoietic stem cells as used by Dingli & Michor [92], and black position markers show the number of cells as used by Gentry et al. [86].

The model is inspired by Dingli and Michor, and therefore the parameter values are based upon their values [92]. However, we have adjusted them to obtain more appropriate saturation levels in agreement with data (see Fig 2 and the reported values in Table C in S1 Appendix). First, the model is calibrated to the situation of no MPN cancer cells (*y*_0i_ = 0 and *y*_1i_ = 0). In this situation, we expect a stable steady state such that the number of HSC is approximately 10^4^ and that of HMC is approximately 10^10^. These choices are compromises between reported values for the number of HSC [78, 86, 88, 89, 92].

From the steady state condition we have the number of dead cells to be ${\text{a}}_{\text{x}}=\frac{d_{x_{0}}x_{0}+d_{x_{1}}x_{1}}{e_{a}s}\;\approx 10^{3}$. We further expect r_x_>d_x0_ + a_x_ and d_x0_ ≪d_x1_. When allowing for MPN development the healthy state becomes unstable when perturbed by the malignant stem cells. Thus, we expect r_y_ >r_x_.

In the final stage the in silico patient will have vanishing hematopoietic cells and the MPN cells will approach a stable steady state with a higher amount of MPN cells than normal hematopoietic cells in the healthy steady state. This is accomplished by choosing all the c-values equal in order to keep the model as simple as possible and the number of parameters as few as possible. Likewise, the parsimonious principle suggests d_y0_ = d_x0_, a_y_ = a_x_ and A_y_ = A_x_.

**Fig 3:**
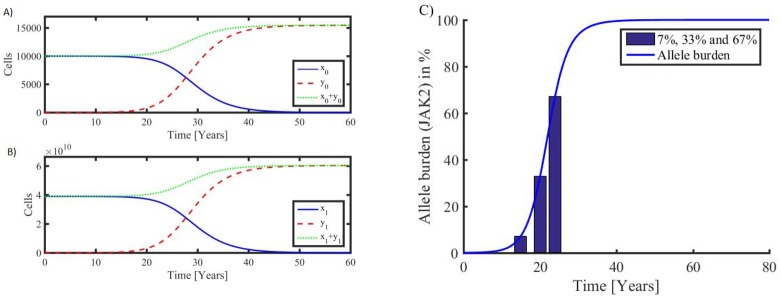
**Left:** Typical development in stem cells (top panel A) and mature cells (bottom panel B). Healthy hematopoietic cells (full blue curves) dominate in the early phase where the number of malignant cells (stipulated red curves) are few. The total number of cells is also shown (dotted green curves). When a stem cell mutates without repairing mechanisms, a slowly increasing exponential growth starts. At a certain stage, the malignant cells become dominant, and the healthy hematopoietic cells begin to show a visible decline. Finally, the composition between the cell types results in a takeover by the malignant cells, leading to an exponential decline in hematopoietic cells and ultimately their extinction. The development is driven by an approximately exponential increase in the MPN stem cells, and the development is closely followed by the mature MPN cells. **Right:** B)The corresponding allele burden (7%, 33% and 67% corresponding to ET, PV, and PMF, respectively) defined as the ratio of MPN mature cells to the total number of mature cells.

The JAK2V617F allele burden has been reported to have median values of 7% (95% CL 2–15%; range 1–39%), 33% (95% CL 20–40%; range 1–92%) and 67% (95% CL 52–95%; range 37–99%) in ET, PV and PMF patients, respectively [19]. It follows that the model output perfectly resamples these dynamic changes in the JAK2V617F mutational load (Fig 3). Additional details are given in the S1 Appendix section.

All these attempts in calibrating the model may simultaneously be considered as validation since they performed successfully. However, the model may be validated further by predicting affected cytokine levels from the inflammatory level. As indicators of the inflammatory level, we refer to those cytokines, which are considered most important in the context of MPNs: IL-1β, IL-1RA, IL-2R, IL-6, IL-8, IL-10, IL-12 and the inflammation biomarker CRP which all turned out to be linearly correlated with the inflammatory level (s).

**Fig 4:**
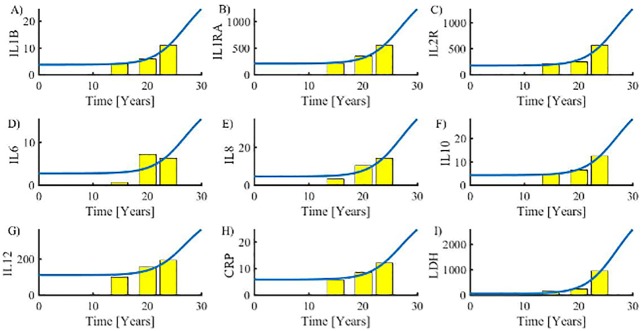
Model validation. Cytokines A) IL-1β, B) IL-1RA, C) IL-2R, D) IL-6, E) IL-8, F) IL-10, G) IL-12 and H) C-reactive proteins (CRP) are approximatively linearly correlated with the inflammatory level s. For the specific cytokines, we have from left to right ‘Normal’, ‘PV’, and ‘PMF’ median values (yellow columns) for comparison based on the predicted inflammatory level s (full blue curve) as a function of time after the first insult. I) Similarly, LDH is correlated with and compared to the total rate of dying cells *DI* = *dx*_0_*x*_0_ + *dx*_1_*x*_1_ + *dy*_0_*y*_0_ + *dy*_1_*y*_1_.

For the specific cytokines (*C*_*i*_) tabulated in the S1 Appendix, we have ‘Normal’, ‘PV’, and ‘PMF’ median values (*m*_*ij*_, where index *i* specifies the cytokine and index *j* refers to ‘Normal’, ‘PV’ and ‘PMF’ states) for each. Then we find *k*_*i*1_ and *k*_*i*2_ such that *m*_*ij*_ = *k*_i1_ *s*_j_+*k*_i2_ where *s*_*j*_ is the value of s at year *t*_*j*_. Similarly, LDH values were demonstrated to be correlated and compared to the total rate of dying cells *DI* = *dx*_0_*x*_0_ + *dx*_1_*x*_1_ + *dy*_0_*y*_0_ + *dy*_1_*y*_1_. The results are summarized in Fig 4 which shows that the model predicts data very well. Only IL-6 seems to be less well predicted.

Disallowing potential mutations and having no MPN-stem cells initially forces the model system into a steady state where solutions are all constant after a possible initial transient event. Introducing a mutation probability introduces a fatal malignant state; the higher the mutation probability is the faster the malignant state develops. A typical scenario is shown in Fig 3A along with a curve of the allele burden development (Fig 3B). The Figure depicts both modeling of the development of MPN from normal HSC and the early MPN diseases stages (ET/PV) to the advanced myelofibrosis stage.

Having a continuous mutation rate, it will take 24 years for the disease to develop to an allele burden of 7% (e.g. ET) and after additional four years the allele burden reaches 33% (e.g. transformation of ET to PV) to become 67% (e.g. transformation of PV to post-PV myelofibrosis) at year 36 after the first stem cell mutation. Disallowing mutations in the model and initially including a single malignant stem cell and no malignant mature cells shifts the allele burden curve by one year to the left on the time axis.

Thus, the mutation of an HSC to MPN SC triggers the disease. Once an MPN stem cell is established the disease can progress without further mutations.

**Fig 5:**
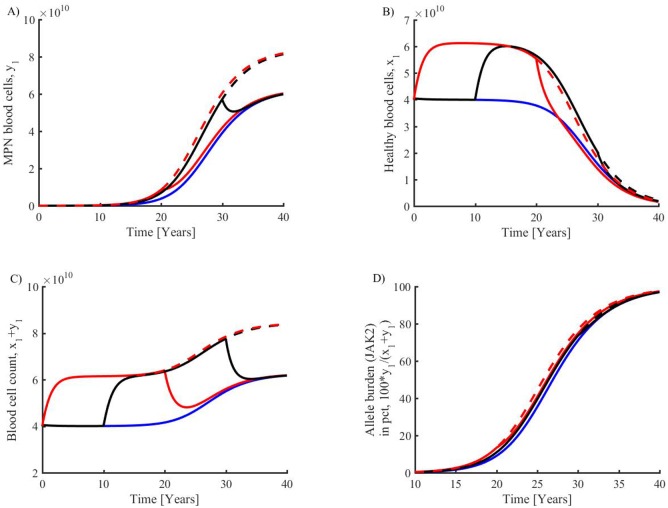
Investigation of increased inflammatory load at various onsets and durations. Blue curve is default parameters corresponding to Fig 3, red dotted is a doubling of inflammatory load, full red curve is a doubling of inflammatory load in year 0–20, then back to default level, black dotted curve is inflammatory doubling from year 10, the full black is inflammatory doubling year 10–30. **Upper:** Increasing inflammatory load has a boosting effect on MPN MC (A) as well as on HMC (B). **Lower:** Displaying the results in terms of the clinically available quantity, total blood cell count, also shows a boosted effect with increasing inflammatory load (C). The allele burden of JAK2 mutated blood cells similarly shows that increased inflammation increases disease development (D). There is a clear effect of MPN promotion with increasing inflammatory load, earlier onset, and exposure time. Lowering inflammatory load makes disease progression less rapid. Maintaining a doubling (red dotted curve) shifts the allele burden curve to the left by two years. Shortening the exposure time of inflammatory load weakens the disease progression. The inflammation has a fast impact on the total number of blood cells, which typically changes by 25% within the first year after doubling or reducing the inflammatory load by 50%.

The baseline inflammatory load (stimulus) is arbitrarily set to 7 pg/ml per day during normal circumstances. It is an exogenous stimulation of the immune system, which leads to an inflammatory level of 3.61 pg/ml, increasing to 3.66 pg/ml in MPNs. This corresponds to a baseline of 700 dead cells (in the hematopoietic steady state) before MPN develops remarkably. A doubling of the baseline inflammatory level is directly affecting the inflammation load (cytokine level) and thereby affecting the rest of the system as dictated by the model equations. In Fig 5 is depicted that shortening the exposure time of inflammation load is associated with deceleration of disease progression.

## Discussion

Chronic inflammation is characterized by persistently activated immune cells, DNA damage, tissue destruction, remodeling and fibrosis [106]. In patients with MPNs, these processes are exemplified by the advanced myelofibrosis stage [4, 29], which accordingly might be considered to develop as the consequence of chronic inflammation in the bone marrow–“the inflamed bone marrow” and “the wound that won’t heal” [4, 29,107,108]. Herein, we for the first time use mathematical modelling to substantiate the concept that MPN progression is facilitated by chronic inflammation and that ET and PV are linked through increasing JAK2V617F allele burden [19] which is destined to happen as time increases without interference. Importantly, we were able to create the inflammation-MPN model based upon current knowledge on the interactions between inflammatory cytokines, hematopoietic stem cells and progenitors, and the bone marrow microenvironment [31–33,35–37,105]. By mathematical modelling of all these interactions, our integrated inflammation-MPN model was created. The model was validated from current data on circulating inflammatory cytokines in MPNs [40,44–46], thereby substantiating inflammation to be a highly potent stimulus for clonal evolution and cancer progression in MPNs. In the context that elevated CRP levels have been shown to be associated with shortened leukemia-free survival in myelofibrosis [42], it is of interest that our model was excellently validated by data on CRP levels in the different MPN disease stages as well.

Mathematical modelling has been used to describe the impact of chronic inflammation and immune deregulation in aging [109] and several diseases, including type 1 diabetes mellitus [110], rheumatoid arthritis [96] and colitis-associated colon cancer [111]. Based upon the known association between respiratory infections and chronic inflammation, Herald described a general model of inflammation [97]. In this model, a system of nonlinear ordinary differential equations was used to describe interactions between macrophages, inflammatory and anti-inflammatory cytokines and bacteria. Though initiated by bacteria as the stimulus to trigger chronic inflammation, their study focused on chronic inflammation in the absence of pathogens as well [97]. Of note, even small changes in parameters of importance for inflammatory cytokine production and macrophage sensitivity to cytokines resulted in dramatically different model behaviors [97]. According to this model chronic inflammation is not triggered when the immune system is functioning properly. However, in patients with a dysfunction of the immune system positive feedback of the inflammatory cytokine network is prone to induce chronic inflammation. Furthermore, if the macrophage population is more sensitive to inflammatory cytokines small perturbations initiated by the inflammation stimulus will also lead to chronic inflammation [97]. In this context, it is intriguing to consider if the inherited genetic predisposition to acquire the JAK2V617F-mutation due to the haplotype 46/1 [112–117], which also confers an increased risk of (other) inflammatory diseases (e.g. Crohns’ disease) [118,119] and /or acquired genetic instability due to sustained chronic inflammation (chronic inflammatory diseases or toxin exposure (e.g. smoking) might further increase the risk of developing MPN—a hypothesis originally proposed by Hermouet et al [33,35]. Importantly, the hypersensitivity of clonal MPN-cells to exogenous and endogenous growth factors and inflammatory cytokines might also more easily lead to a chronic inflammatory state–similar to the increased sensitivity of the macrophage population leading to chronic inflammation in the Herald model and also implemented in the Hermouet model, implying an enhanced myelomonocytic response to cytokine stimulation [33,35].

In the Herald model and the model described by Nielsen et al in regard to type 1 diabetes mellitus, the macrophages constituted an important compartment [97,110]. The monocyte-macrophage cell lineage is of major importance in the context of inflammation and cancer development. In our MPN-inflammation model bone marrow macrophages are also of utmost importance—both in regard to release of inflammatory cytokines, but also in regard to the development of myelofibrosis. Thus, in MPNs the monocyte-macrophage cell—together with the megakaryocyte (MK) cell lineage—are considered to be responsible for the development of myelofibrosis by the release of a number of growth factors and inflammatory cytokines that stimulate fibroblast proliferation [36,120,121]. The “Herald Model” is in several aspects equivalent to our model when considering substituting “bacteria”in the “Herald Model” by any noxious inflammatory stimulus. In fact, we implement yet another cell lineage—the MKs—as the source of a continuous release of products that stimulate the vicious inflammation circle, implying ultimately the development of cancer—the MPNs. As previously outlined, our mathematical modelling of the concept of chronic inflammation in MPNs is also supported by the elegant model described by Hermouet and co-workers [33,35], in which the *JAK2* 46/1 haplotype was proposed as a marker of inappropriate myelomonocytic response to cytokine stimulation, leading to increased risk of inflammation, myeloid neoplasms, and impaired defense against infection [33]. Indeed, the Hermouet model for chronic inflammation [33,35] fits exceedingly well with the Herald model of general inflammation [97] and our mathematical modelling of MPNs as “A Human Inflammation Mode for Cancer Development [29–32]. In this regard, chronic inflammation and immune deregulation in MPNs might act as a trigger for later development of AML and MDS in line with the known association of inflammatory signaling and cancer [24–27]. The above models are additionally supported by the hypothetical model by Takizawa et al. (2010) [122], describing how chronic inflammatory processes might impinge on hematopoiesis, potentially fostering hematopoietic stem cell diseases, including MPNs. By inducing high proliferation of most HSCs, chronic inflammation might give rise to both exhaustion of the HSC pool and an even greater risk to accumulate genetic alterations in HSCs. Furthermore, by inflammatory stimuli from the bone marrow microenvironment these genetically altered HSCs might be rescued or “cancer cell niche” for later development of a hematological cancer [122].

The perspectives of our study are several. In the context that myelomonocytic cells (granulocytes, macrophages, monocytes) and MKs are all deeply involved in cancer development and progression [123,124], chronic inflammation is associated with premature atherosclerosis (atherothrombosis) [21–23, 29,30], in which both platelets and monocytes are highly important (monocytes a link between atherosclerosis and cancer [28]) and platelets are intimately involved in the metastatic process in cancer [124]—and likely in MPNs as well [125]—the avenue is opened for studying all these aspects by using mathematical modelling of current knowledge of the impact of chronic inflammation and immune deregulation in patients with MPNs. Ultimately, mathematical modelling may also be able to substantiate which agents to be used in MPNs in order to induce “minimal residual disease”[125–129] and the importance of early intervention with agents that directly target both the malignant clone (interferon-alpha2) [126–129] and the inflammatory process (JAK1-2 inhibition with e.g. ruxolitinib) [130].

In conclusion, we have for the first time applied mathematical modelling as a tool to deliver the proof of concept that chronic inflammation is closely linked to the development of the MPNs—myeloproliferative cancers which today are considered to be “chronic inflammatory diseases”, in which chronic inflammation may be a driving force for clonal expansion and ultimately the development of AML [4, 29–32,39]. Studies are ongoing to elucidate the above perspectives by mathematical modelling. In this regard, mathematical modelling of resolution of inflammation may be highly important [98] and useful to support the decision-making which agents to use in the future for patients with MPNs in order to induce minimal residual disease and hopefully cure.

## Supporting information

S1 Appendix (DOCX)
