# Supplementary material for: Mathematical modelling as a proof of concept for MPNs as a human inflammation model for cancer development
Source: PLoS One. 2017 Aug 31;12(8):e0183620. doi: 10.1371/journal.pone.0183620 (PMC5578482; doi:10.1371/journal.pone.0183620)
Supplement: S1 Appendix — (DOCX) [file pone.0183620.s001.docx]

**S1 Appendix**

**The model:**

For pedagogical reasons we divide the presentation of the model into two phases, one only including cell population dynamics ignoring niche feedback and inflammatory feedback related to cell mortality, in the next step followed by an elaboration of this including the omitted elements. The first phase is essentially the model presented by Dingli & Michor [92] whereas the second phase represents our natural extension.

The basic model without niche feedback and inflammatory feedback related to cell mortality reads,

| $\frac{dx_{0}}{dt}=\left( r_{x}-a_{x}-d_{x0} \right)x_{0}-r_{m}x_{0}$, | $x_{0}\left( 0 \right)=x_{0i}$, | $\text{[}x_{0}\text{=HSC]}$, | (1) |
| --- | --- | --- | --- |
| $\frac{dx_{1}}{dt}=a_{x}A_{x}x_{0}-d_{x1}x_{1}$, | $x_{1}\left( 0 \right)=x_{1i}$, | $\text{[}x_{1}\text{=HMC]}$, | (2) |
| $\frac{dy_{0}}{dt}=\left( r_{y}-a_{y}-d_{y0} \right)y_{0}+r_{m}x_{0}$, | $y_{0}\left( 0 \right)=y_{0i}$, | $\text{[}y_{0}\text{=}\text{MPN }\text{SC]}$, | (3) |
| $\frac{dy_{1}}{dt}=a_{y}A_{y}y_{0}-d_{y1}y_{1}$, | $y_{1}\left( 0 \right)=y_{1i}$, | $\text{[}y_{1}\text{=}\text{MPN }\text{MC]}$, | (4) |

where $x_{0i}, x_{1i}, y_{0i},$ and $y_{1i}$ denotes the initial number of the respective cells. We emphasize the three possible interpretation of the mutation part of the model: 1) the term $r_{m}x_{0}$ is interpreted as a stochastically variable and$y_{0i}=0$; 2) it is interpreted as a non-zero continuous variable and$y_{0i}=0$; or 3) it is interpreted as zero (i.e. $r_{m}=0$) and $y_{0i}=1$. In the present work we have mainly adopted the third interpretation.

The full model including the inflammatory feedback related to cell mortality and niche feedback becomes,

| $\frac{dx_{0}}{dt}=\left( r_{x}\varphi_{x}s-d_{x0}-a_{x} \right)x_{0}-r_{m}sx_{0}$, | $x_{0}\left( 0 \right)=x_{0i}$, | $\text{[}x_{0}\text{=HSC]}$, | (5) |
| --- | --- | --- | --- |
| $\frac{dx_{1}}{dt}=a_{x}A_{x}x_{0}-d_{x1}x_{1}$, | $x_{1}\left( 0 \right)=x_{1i}$, | $\text{[}x_{1}\text{=HMC]}$, | (6) |
| $\frac{dy_{0}}{dt}=\left( r_{y}\varphi_{y}s-d_{y0}-a_{y} \right)y_{0}+r_{m}sx_{0}$, | $y_{0}\left( 0 \right)=y_{0i}$, | $\text{[}y_{0}\text{=}\text{MPN }\text{SC]}$, | (7) |
| $\frac{dy_{1}}{dt}=a_{y}A_{y}y_{0}-d_{y1}y_{1}$, | $y_{1}\left( 0 \right)=y_{1i}$, | $\text{[}y_{1}\text{=}\text{MPN }\text{MC]}$, | (8) |
| $\frac{da}{dt}= d_{x0}x_{0}+d_{y0}y_{0}+d_{x1}x_{1}+d_{y1}y_{1}-e_{a}as$, | $a\left( 0 \right)=a_{i}$, | $\left[ a\text{=dead cells} \right],$ | (9) |
| $\frac{ds}{dt}= r_{s}a-e_{s}s+\text{inf}\text{lammatory load}$, | $s\left( 0 \right)=s_{i}$, | $[s\text{=inflammatory level}]$, | (10) |

Here the feedback from the niches in the bone marrow affecting the self-renewal rates are represented by decreasing Hill-functions in the number of stem cells,

$$\begin{matrix} \varphi_{x}(x_{0},y_{0})=\frac{1}{1+{(c_{xx}x_{0}+c_{xy}y_{0})}^{2}} & \text{and} & \varphi_{y}(x_{0},y_{0})=\frac{1}{1+{(c_{yx}x_{0}+c_{yy}y_{0})}^{2}} \end{matrix}$$

The coupling parameters $c_{ij}$ represent the strength by which the amount of the $j$’th stem cell type affects the growth of the $i$’te stem cell type. These expressions are in analogy with Gentry et al [86] but differentiate slightly from the Michaelis-Menten expressions used by Michor et al 100] and Stiehl et al. [88,89]. Furthermore, an age dependence may be included in the mutation part of the model as a constant age-dependent function multiplied on the term $r_{m}sx_{0} \left[ 100 \right].$The numerical values of the model parameters are chosen to fulfil hematopoiesis (steady state) when r_m_=0 and no MPN cancer cells are present.

**Model calibration and validation:**

The model is inspired by Dingli & Michor [92]. However, we have adjusted some of these to obtain more appropriate saturation levels in agreement with data and clinical experiences. First, the model is calibrated to the situation of no MPN cancer cells, i.e. $y_{0i}=0$ and $y_{1i}=0$. In general at steady state,

$$x_{1}=\frac{a_{x}A_{x}}{{dx}_{1}}x_{0},$$

$$y_{1}=\frac{a_{y}A_{y}}{{dy}_{1}}y_{0},$$

$$a=\frac{-\frac{e_{a}}{e_{s}}BIS+\sqrt{{(\frac{e_{a}}{e_{s}}BIS)}^{2}+4r_{s}\frac{e_{a}}{e_{s}}D}}{2r_{s}\frac{e_{a}}{e_{s}}},$$

and

$$s=\frac{{k(r}_{s}a+BIS)}{e_{s}}$$

where *BIS* denotes the baseline exogenous inflammation load and

$D={dx}_{0}x_{0}+{dx}_{1}x_{1}+{dy}_{0}y_{0}+{dy}_{1}y_{1}$ is an abbreviation for the total of dying cells per time.

From the above, the parameter values have to fulfil these equations at *t=0* if we assume start in a healthy steady state, e.g. $\frac{{a_{x}A}_{x}}{d_{x1}}=\frac{x_{1}}{x_{0}}\approx{10}^{7}$. Thus, the involved parameters have been chosen accordingly. We expect the number of HSC to be approximately 10^4^ and that of HMC to be approximately 10^10^ in the healthy steady state. These choices are compromises between reported values: Gentry & Jackson [86] use 10^4^, respectively 10^10^, Stiehl et al. [88,89] use 8·10^7^ per kg, respectively 2·10^9^ per kg, Dingli & Michor [92] use 2·10^4^ , respectively 10^12^; and Haeno et. al. [78] use 5·10^5^ for the number of HSC, see Table 1 below. From the steady state condition we also have the number of dead cells to be $a=\frac{d_{x_{0}}x_{0}+d_{x_{1}}x_{1}}{e_{a}s}\approx700.$

Regarding the inflammatory level (s) we have chosen the value 3.61 pg/mL. The inflammatory level s is an abstraction. Hence, the values are not known and only a relative change in s matters in the model.

**Tables**

**Table A. Inflammatory cytokines /C-Reactive Protein**

| **Disease/Cytokine** | | **Normal** | **ET** | **PV** | **PMF** | **Reference** |
| --- | --- | --- | --- | --- | --- | --- |
| Il-1beta (pg/ml) | 4.0 | | ND | 5.9 | 10.8 | 44,45 (PV,PMF) |
| IL-1RA (pg/ml) | 203.2 | | ND | 342.4 | 552.0 | 44,45 (PV,PMF) |
| IL-2R (pg/ml) | 216.9 | | ND | 250.4 | 555.9 | 44,45 (PV,PMF) |
| IL-6 (pg/ml) | 0.6 | | ND | 11.8 | 6.2 | 44,45 (PV,PMF) |
| IL-8 (pg/ml) | 3.2 | | ND | 10.2 | 14.3 | 44,45 (PV,PMF) |
| IL-10 (pg/ml) | 4.8 | | ND | 6.5 | 12.5 | 44,45 (PV,PMF) |
| IL-12 (pg/ml) | 100.2 | | ND | 156.0 | 191.8 | 44,45 (PV,PMF) |
| C-Reactive Protein  (CRP) | < 2.9 | | 5.6 | 8.5 | 12.1 | Unpublished series of CRP at the time of diagnosis in 27 ET patients , 38 PV patients and 42 PMF patients |
| LDH (U/L) | 152 | | ND | 226 | 935 | 19 |

**Table B. Default Parameters.**

| **Parameter** | **Value** | **Units** | **Parameter** | **Value** | Units |
| --- | --- | --- | --- | --- | --- |
| r_x_ | 8.7·10^-4^ | day^-1^ | r_y_ | 1.3·10^-3^ | day^-1^ |
| a_x_ | 1.1·10^-5^ | day^-1^ | a_y_ | a_x_ | day^-1^ |
| A_x_ | 4.7·10^13^ | - | A_y_ | A_x_ | - |
| d_x0_ | 2·10^-3^ | day^-1^ | d_y0_ | d_x0_ | day^-1^ |
| d_x1_ | 129 | day^-1^ | d_y1_ | d_x1_ | day^-1^ |
| c_xx_ | 7.5·10^-5^ | - | c_yx_ | c_xx_ | - |
| c_xy_ | c_xx_ | - | c_yy_ | c_xx_ | - |
| e_s_ | 2 | day^-1^ | r_s_ | 3·10^-4^ | day^-1^ |
| r_m_ | 0 or 2·10^-8^ | day^-1^ | e_a_ | 2·10^9^ | day^-1^ |
| Inflammation  (pg/ml) | 7 | day |  |  |  |

r_x_ = self renewal rate of HSC,

a_x_ = proliferation rate of HSC,

A_x_ = multiplication factor which describe the ration of the rate of produced HMC and a_x_ lumping the hematopoietic progenitor cell dynamics into one factor A,

d_x0_ = dead rate of HSC,

d_x1_ = dead rate of HMC,

c_xx_ = factor describing the strength by which the HSC inhibits their own self renewal,

c_xy_ = factor describing the strength by which the MPN SC inhibits self renewal of the HSC,

r_y_ = self renewal rate of MPN SC,

a_y_ = proliferation rate of MPN SC,

A_y_ = multiplication factor which describe the ration of the rate of produced MPN MC and a_y_ lumping the MPN progenitor cell dynamics into one factor A,

d_y0_ = dead rate of MPN SC,

d_y1_ = dead rate of MPN MC,

c_yx_ = factor describing the strength by which the HSC inhibits self renewal of the MPN SC,

c_yy_ = factor describing the strength by which the MPN SC inhibits their own self renewal,

e_s_ = elimination rate of cytokines (more precisely the abstract quantity s),

r_s_ = rate by which the dead cells up-regulate the amount of phagocytic cells per dead cell,

e_a_ = elimination rate of dead cells per cytokine (dead cells is down-regulated as a second order elimination process given by *-e_a_·a·s*),

r_m_ = effective MPN mutation rate

**Table C**: **Hematopoietic Stem Cell** (**HSC), MPN Stem Cell (MPN SC), Hematopoietic Mature Cell (HMC), and MPN Mature Cell (MPN MC ) counts under various circumstances with references.** Values are our calculated values from the values given in [88,89] in counts per kg assuming a weight of 80 kg [89]. The two last rows represent our expected values, which have been used for validation in the Figures. Following Figure 1 illustrates model prediction and the validation by data.

| **Ref.** | **HSC** | **MPN SC** | **HMC** | **MPN MC** | **a** | **s** |
| --- | --- | --- | --- | --- | --- | --- |
| [86] | 10^4^ | >10^10^ | 10^10^ |  |  |  |
| [88,89] | 6.4·10^9^ |  | 1.6·10^11^ |  |  |  |
| [78] | 5·10^5^ |  |  |  |  |  |
| [92] | 2·10^4^ | >10^5^ | 10^12^ | >10^13^ |  |  |
| Our “no cancer values” | 1.01·10^4^ | 0 | 4·10^4^ | 0 | 699.9 | 3.61 |
| Our MPN values | 0 | 1.56·10^4^ | 0 | 6.25·10^10^ |  |  |

**Table D. Initial values.**

| **Variable** | x_0_  (HSC) | x_1_  (HMC) | y_0_  (MPN SC) | y_1_  (MPN MC) | a  (Dead Cells) | s  (Inflammatory level) |
| --- | --- | --- | --- | --- | --- | --- |
| **Initial value** | 1.01·10^4^ | 3.84·10^10^ | 0 | 0 | 699 | 3.61 pg/ml |

**Additional Graphs**

**Figure S1**

| **** | **** |
| --- | --- |
|  | **** |

**Figure S1**. **Upper left:** HSC (x_0_), MPN SC (y_0_), HMC (x_1_), and MPN MC (y_1_) predicted curves versus time. **Upper right:** Shows the logarithm of the corresponding curves shown to the left. Strait sections of curves are when the original curves change exponentially. **Lower left:** Green curves are the amount of dead cells (a) and magenta is the inflammatory level. **Lower right**: The JAK2V617F allele burden curve with 7% (ET), 33% (PV), and 67% (PMF) emphasized by blue columns.

**Sensitivity Analysis**

The analysis enables to analyze those parameters which affect the output mostly and at the same time addressing how they are affected if they were varied one at a time keeping the rest fixed at their nominal values. We choose 10% variation in the values of parameters and simulate the effect on cell counts, time intercept (i.e. time from 0 until x_0_=y_0_) and acquired percentage of the JAK2V617F allele burden. The parameters which have the greatest impact upon the output of the model are illustrated

in Figure 2 below. These include self-renewal rates and death rates of HSC and MPN SC as well as $c_{x}$ and $c_{y}$.

|  | **Decreased value** | **Increased value** | **Decreased value** | **Increased value** |
| --- | --- | --- | --- | --- |
| **r_x_** | **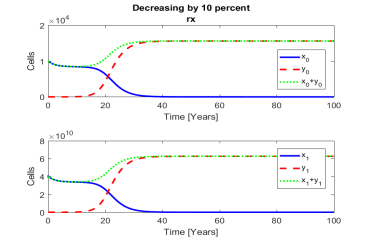** | **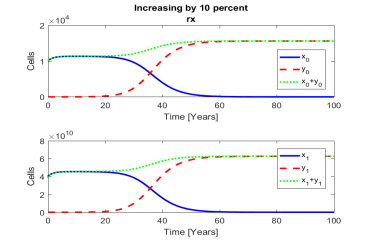** | **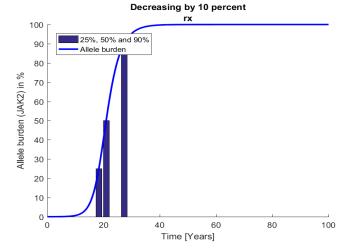** | **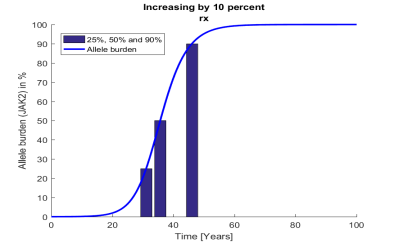** |
| **r_y_** | **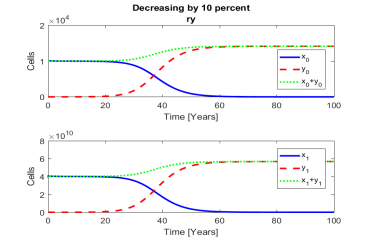** | **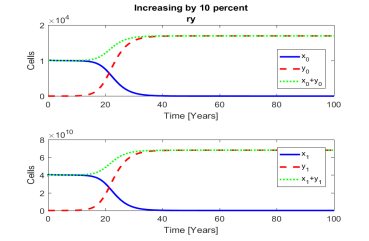** | **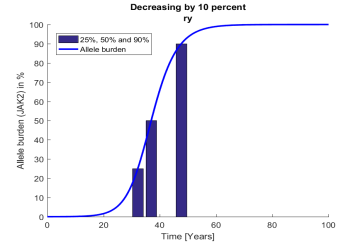** | **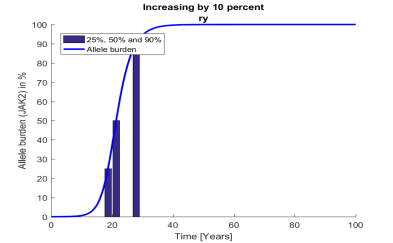** |
| **d_x0_** | **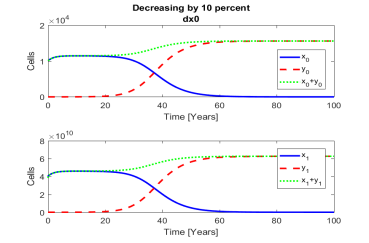** | **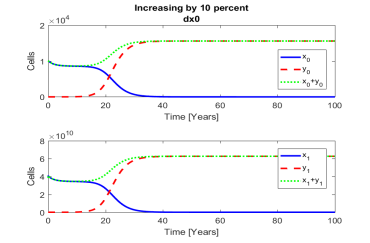** | **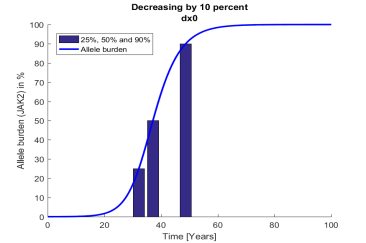** | **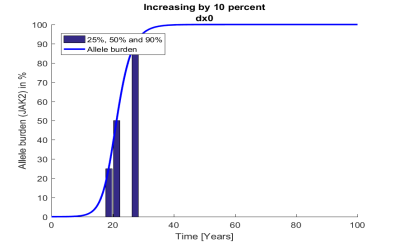** |
| **d_y0_** | **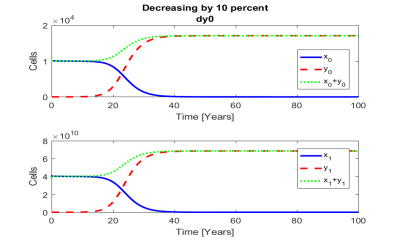** | **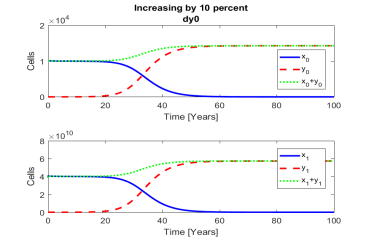** | **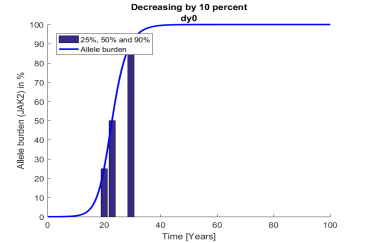** | **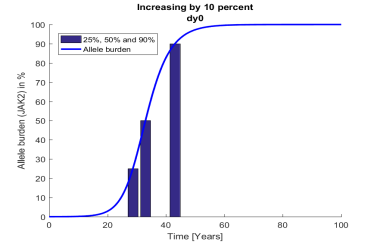** |
| **c_x_** | **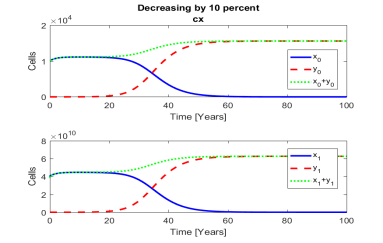** | **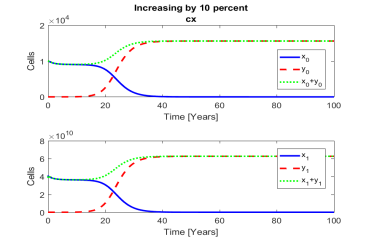** | **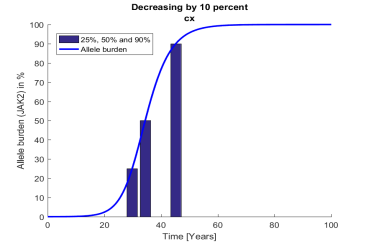** | **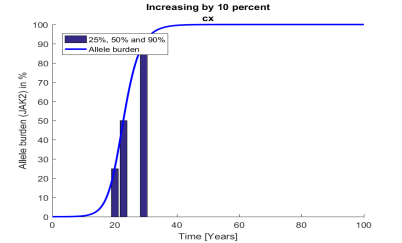** |
| **c_y_** | **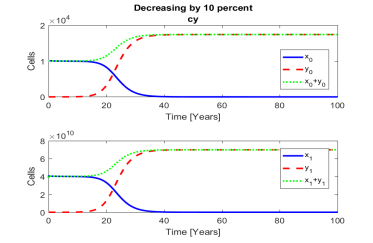** | **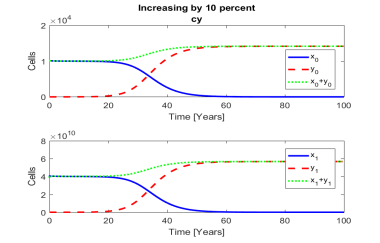** | **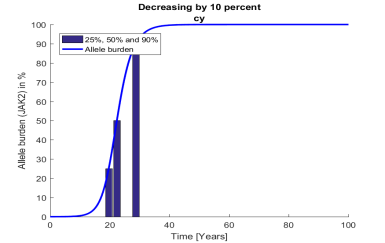** | **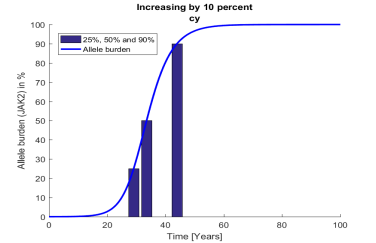** |
| **e_s_** | **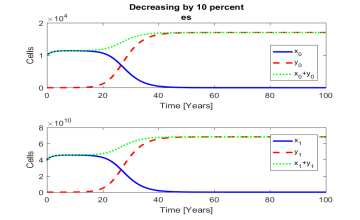** | **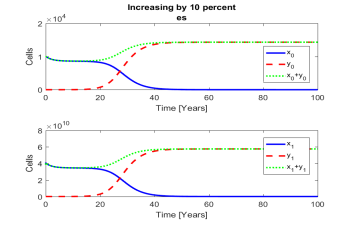** | **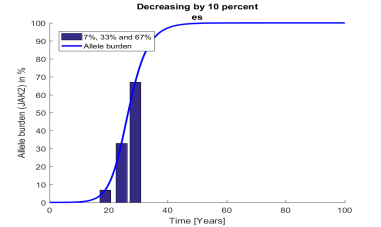** | **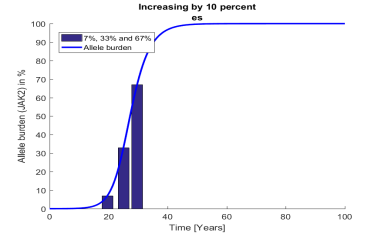** |
| **I** | **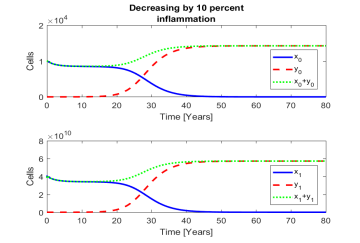** | **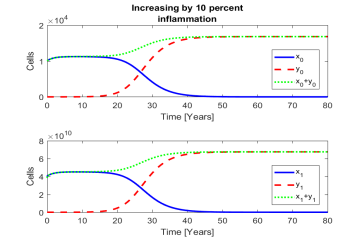** | **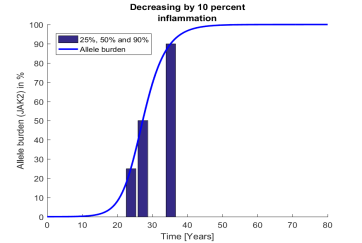** | **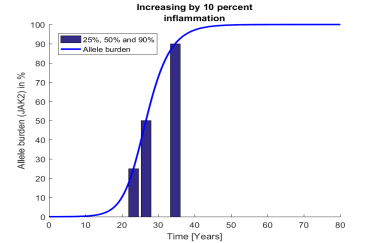** |

**Figure S2**. Sensitivities for selected parameters. 1^st^ column shows the parameters, 2^nd^ column shows how the output curves of the cell numbers are changed when the parameter is decreased by 10%, 3^rd^ column shows the corresponding curves for a 10% increase in the parameter, and the two last columns the corresponding values for the JAK2V617F allele burden.

From Figure S2 we observe which of the parameters have the most pronounced impact upon the lag-time. The 1^st^ and 3^rd^ columns show the effect of decreasing the parameter values by 10%. Here $r_{y}$ and $d_{x0}$ increase the time intercept equally well but more markedly than other parameters. In addition, by increasing parameter values (in the 2^nd^ and 4^th^ column), $r_{x}$ has the most pronounced effect on lag-time followed by $c_{y}$ and $d_{y0}$ which influence the output almost similarily.

A more clear picture of change in lag-time can be seen from the following bar charts in Figure S3.

**Figure S3:** Bars above and below 1 show increase and decrease in duration to time-intercept (i,.e. until x_0_=y_0_) respectively. Here, parameters labels the x - axis and average values w.r.t time as y - axis

In addition to lag-time some parameters also change cell counts especially number of MPN cells, $y_{0}$ and $y_{1}$. This is illustrated in Figure S4.

**Figure S4.** Bars above and below 1 show increase and decrease in cell counts, respectively, when a parameter is varied (values equal to 1 are cell counts for the default parameter values). Parameters are shown along the x-axis and the average cell counts along the y-axis. For each parameter four columns are shown; The first (D:y0 in light blue) shows the effect on y_0_ when the parameter is decreased by 10%, the second (I:y0 in brown) shows the effect on y_0_ when the parameter is increased by 10%, the third (D:y1 in green) shows the effect on y_1_ when the parameter is decreased by 10%, and fourth (I:y1 in dark blue) shows the effect on y_1_ when the parameter is increased by 10%.
